# Protein intakes for preterm infants, and the need for a multi-nutrient holistic approach

**DOI:** 10.1038/s41390-024-03453-4

**Published:** 2024-08-10

**Authors:** Nicholas D. Embleton, Chris H. P. van den Akker

**Affiliations:** 1 Neonatal Unit, Royal Victoria Infirmary, Population Health Sciences Institute, Newcastle University, Newcastle upon Tyne, United Kingdom; 2 Department of Pediatrics – Neonatology, Amsterdam UMC, Emma Children’s Hospital, University of Amsterdam, Amsterdam, The Netherlands; 3 Amsterdam Reproduction & Development Research Institute and Amsterdam Gastroenterology Endocrinology & Metabolism Research Institute, Amsterdam, The Netherlands

In this edition of Pediatric Research, Das et al. present the results of a systematic review of high versus low dietary protein intake in neonates born preterm.^1^ The review assessed the potential benefits or risks of higher protein intakes during the first month of life reported in 44 studies and concluded that whilst growth prior to discharge was often greater, there were no longer term growth benefits and higher intakes may represent a risk for neonatal metabolism and later neuro-disability. However, the level of certainty for most findings was low, or non-significant, and the number of participants in most studies was small. Nevertheless, many have previously expressed concerns that currently recommended protein intakes may be too high. Most neonatal randomised controlled trials (RCTs) of nutritional interventions have been powered on short-term outcomes such as anthropometry and there are few RCTs powered to detect a meaningful difference in functional outcomes such as infant neurodevelopment.

The PEPaNIC study was a ground-breaking RCT conducted in infants and children and demonstrated the risks of inappropriately high nutrient intakes to acutely critically ill patients.^2^ Whilst the study did not recruit preterm infants, the study highlights that nutrient intake in excess of dietary requirement and/or metabolic capacity can be harmful, especially for amino acid intakes. This is critically important for preterm infants where short-term biochemical derangement such as hyper- and hypoglycaemia, hypokalaemia and hypophosphatemia are common, and where predicted protein requirements are, in part, based on factorial methods. A 2018 multi-society guideline on parenteral nutrition (PN) recommended to start parenteral amino acids immediately after birth in preterm neonates and to increase the next day to 2.5–3.5 g/kg/day accompanied by sufficient non-protein energy (>65 kcal/kg/day) and micronutrients.^3^ The recent ESPGHAN position paper on enteral nutrition^4^ strongly recommends a total protein intake of 3.5–4.0 g/kg/day, which may be increased to 4.5 g/kg/day where growth is slower than anticipated and where other correctable causes of growth faltering such as suboptimal energy intakes or electrolyte abnormalities have been addressed, but note that the maximum parenteral amino acid intakes are still limited at 3.5 g/kg/day.

The review of Das et al. focuses on protein intakes and lumps studies administering parenteral amino acids and enteral protein together.^1^ We do not consider these to be comparable. Humans have an essential requirement for amino acids rather than protein, notwithstanding the critical functional role of whole proteins in human breast milk such as lactoferrin. The fetus receives amino acids via the placenta in an amount far greater than that needed for lean mass accretion alone; transplacental uptake is around 3.5 to 4.0 g/kg/day, while accretion is only half.^5^ Amino acids provided in excess of needs are oxidised for energy. Most studies of PN are conducted in the first two weeks of life when PN is used as a ‘bridge’ to full enteral nutrition (EN), whilst most studies of EN enrol infants later. Therefore, studies of PN and EN cover different postnatal stages of development, with changing hepatic and renal function and maturation including urea synthetic and urinary disposal capacity. This may partly explain the higher tolerance for enteral protein than for parenteral amino acids. Also, when PN is administered, it is not feasible to provide similarly high energy intakes, as parenteral lipid provision is limited at 3.0–4.0 g/kg/day. Thus, while the optimal energy/protein ratio is around 30–35 kcal per g of administered protein, this cannot be achieved via PN in the upper parenteral amino acid intake ranges, partly also explaining the higher serum urea concentrations commonly observed. Furthermore, splanchnic metabolism represents a much greater proportion of energy and amino acid turnover when infants are fed enterally, whereas during PN splanchnic metabolism is minimal. Splanchnic tissues may also adapt and correct any potential imbalances in amino acid uptake arising from suboptimal ‘protein quality’. However, during PN, the liver is bypassed at first pass, and any suboptimal amino acid composition of PN is not corrected. Protein requirements and tolerance are therefore lower when infants are fed using PN compared to EN. All these reasons make us challenge the methodology of combining intakes from PN and EN.

Refeeding syndrome in PN fed newborn infants, especially those born following restricted fetal growth, has been widely observed in recent years.^6,7^ It includes the cardinal hallmarks of hypophosphatemia and hypokalaemia, which may result in worse respiratory functioning, sepsis susceptibility, and hyperglycemia.^8^ In some cases, this represents ‘true’ cases of refeeding syndrome which potentially necessitate lower starting amounts of amino acids, and more gradual increments in provision to avoid overloading cellular autophagy. In other cases, low serum concentrations of potassium and phosphate may be due to inappropriate formulation of PN solutions with inadequate electrolyte provision. In practice, it is probably a combination of both aspects.

Historically, there were concerns that provision of sodium in the first few postnatal days may impair the early water loss needed as extracellular fluid volume contracts after birth. Excess sodium and fluid intakes in this period may worsen respiratory function. However, sodium-free PN restricts the ability to provide disodium-glycerophosphate, the contemporary primary formulation of parenteral phosphate. Similarly, the incidence of early hypokalaemia can be reduced by providing higher intakes of potassium.^9^ A few decades ago, when amino acids were withheld, non-oliguric hyperkalaemia was frequently encountered due to cellular catabolism. However, the fear of early hyperkalaemia may still be deeply rooted among clinicians, despite this being uncommon once infants are supplemented with amino acids and are directed into anabolism.^10^ The optimal ratio of calcium to phosphate differs between PN and EN, with equimolar intakes now recommended for PN, whereas for EN higher intakes of calcium than phosphate are needed because of incomplete calcium absorption. The widespread availability of more balanced PN solutions and closer attention to monitoring means that hypokalaemia and hypophosphatemia are now less common in institutions that pay attention to these potential disturbances including early measurement and treatment of phosphate concentrations. So-called PN starter bags, i.e., low-electrolyte PN bags, are no longer recommended because of risks of refeeding-like syndrome. We therefore feel it would be inappropriate to restrict amino acid intake simply because of these biochemical abnormalities, especially as many cases can be prevented and/or relatively easily treated.

The amino acid composition of breastmilk uniquely meets the needs of healthy term infants; bovine protein contains a different balance of amino acids and needs to be provided in a higher dose to compensate for relative shortage of certain (conditionally) essential amino acid. The optimal composition of amino acids in PN has not been widely studied and it remains unclear what the reference ranges for plasma amino acids should be, particularly because fetal blood concentrations differ from healthy infants ex-utero.^11^ Several PN studies show a wide range of concentrations for both essential and non-essential amino acids, raising the possibility that certain amino acids may be provided in excess and could be neuro-toxic, or conversely that they may be rate limiting for lean mass accretion. Regardless of the optimal plasma amino acid profile, currently available PN solutions will not provide a ‘protein quality’ as high as human milk protein. In addition, most studies do not consider the hydration factor of amino acids in PN, thereby overestimating true protein intakes. Around 15% of the weight of an individual amino acid in PN is lost in the form of water once polymers are formed as protein.^12^ Therefore, a higher intake of amino acids (as nitrogen) in PN might be needed to match the quality and quantity of protein provided by human milk.

Whilst there are few large RCTs with long term cognitive outcome, there are data in preterm infants to show that neonatal macronutrient intakes are associated with outcomes in adolescents., Combined with observational follow-up studies, the idea was supported that higher intakes are beneficial, despite the largest study was conducted in infants born over 30 years ago. Furthermore, the higher protein intakes were typically ~3 g/kg/day rather than over 3.5–4.0 g/kg/day, so the impact of higher intakes require further study. In addition, even if enteral protein intakes >3.5 g/kg/day result in greater growth rates on the NICU, there is scant evidence this improves later cognition, and there are increasing concerns about whether higher infant protein intakes increase the risk of later adverse cardio-metabolic outcomes. Furthermore, position papers and recommendations for intakes are provided assuming a heterogenous group of 'healthy growing preterm infants', yet macronutrient needs vary considerably between 24 weeks and 34 weeks gestation; more premature infants probably require higher intakes.

The paper by Das et al.^1^ is a useful reminder that we have much to learn, and adequately powered trials of nutrient intakes with long term functional outcomes are urgently needed. We remain confident that protein ‘equivalent’ intakes of 3.0–3.5 g/kg/day via PN and 3.5–4.0 g/kg/day via EN are safe and beneficial for most healthy preterm infants based on currently available evidence; that some extremely preterm infants may benefit from higher intakes, and that critically sick infants are likely to require much lower intakes acutely.^13^ However, solely focusing on or changing a single ‘ingredient’ of nutrition, such as increasing amino acid intake, is an outdated approach, whereas a more ‘holistic approach’ of assessing all related nutrients is warranted. An adverse outcome of increased protein intake may not be due to the protein but may also be caused by other disturbances such as refeeding syndrome. If all related issues are addressed properly, then higher intakes ranges might be beneficial. We need closer attention to detail with careful monitoring of biochemical abnormalities and growth, more innovation of PN solutions and greater knowledge of which babies benefit most from high dose breast milk fortification. Whilst we feel current recommendations offer the optimal balance between the risks of brain harm caused by inadequate macronutrient intakes, and the short- and long-term metabolic risks of higher amino acid and protein intakes, we fully support the need for future high-quality RCTs that compare higher and lower intakes than current recommendations and simultaneously supply and assess related nutrients.
